# Loss of Chromosome 3q Is a Prognostic Marker in Fusion-Negative Rhabdomyosarcoma

**DOI:** 10.1200/PO.23.00037

**Published:** 2023-09-22

**Authors:** Carina A. Dehner, Robert C. Bell, Yang Cao, Kevin He, John S.A. Chrisinger, Amy E. Armstrong, Marielle Yohe, Jack Shern, Angela C. Hirbe

**Affiliations:** ^1^ Department of Pathology and Immunology, Washington University School of Medicine, St Louis, MO; ^2^ Department of Pathology/Dermatopathology, Indiana University, Indianapolis, IN; ^3^ Department of Pathology, University of Michigan, Ann Arbor, MI; ^4^ Division of Oncology, Washington University School of Medicine, St Louis, MO; ^5^ Division of Pediatric Hematology/Oncology, Washington University School of Medicine, St Louis, MO; ^6^ Center for Cancer Research, National Cancer Institute, National Institutes of Health, Bethesda, MD; ^7^ Center for Cancer Research, National Cancer Institute, Bethesda, MD

## Abstract

**PURPOSE:**

Soft tissue sarcomas (STS) are rare mesenchymal neoplasms that frequently show complex chromosomal aberrations such as amplifications or deletions of DNA sequences or even whole chromosomes. We recently found that gain of chromosome (chr) 8 is associated with worse overall survival (OS) in STS as a group. We therefore aimed to investigate the overall copy number profile of rhabdomyosarcoma (RMS) to evaluate for prognostic signatures.

**METHODS:**

Fluorescence in situ hybridization (FISH) testing was performed on a cohort of STS to assess for chr8 gain. Copy number variation (CNV) data from the National Cancer Institute were analyzed to assess for prognostically significant CNV aberrations in *FOXO1* fusion–negative (FN)- versus fusion–positive (FP)-RMS. FISH testing was performed on a cohort of FN-RMS to assess for chr3q loss and correlate with outcomes.

**RESULTS:**

Chr8 gain is a highly prevalent CNV in embryonal RMS and shows slightly improved prognosis. Meanwhile, loss of chr3q was associated with worse outcome in FN-RMS compared with FP-RMS.

**CONCLUSION:**

The pathogenesis of STS including FN-RMS remains poorly understood, emphasizing the need for new therapeutic advances and adequate risk stratification. Our data demonstrate that loss of chr3q is associated with poor OS in FN-RMS, supporting it as an important tool for risk stratification.

## Teaser

We show that loss of chr3q is associated with worse outcomes in fusion-negative (FN)-RMS compared with fusion-positive (FP)-RMS, suggesting that this chromosomal abnormality could be a prognostic marker for (FN) RMS.

## INTRODUCTION

Rhabdomyosarcoma (RMS) represents a heterogeneous group of rare, aggressive mesenchymal neoplasms that can present at any age. Despite more aggressive strategies and refinement of treatment approaches, including multimodal chemotherapy, surgery, and/or radiation therapy, which improved survival outcomes for patients with low- and intermediate-risk diseases, those with distant metastases continue to do poorly. RMS has traditionally been separated into two major subgroups—the presence of a *FOXO1* fusion is associated with a worse prognosis, whereas those without a *FOXO1* fusion have been shown to confer a better prognosis.^1^ Recent advances, however, show that this separation is too simplified. In particular, cases within the fusion-negative (FN) RMS group can be further stratified on the basis of additional histologic and molecular features. For example, embryonal RMS (ERMS) should be separated from spindle cell/sclerosing RMS (ssRMS) given that the latter is histologically distinct and portends a significantly worse prognosis. In addition, there are molecular differences with ERMS having several distinct driver mutations within the RAS pathway genes and other cancer genes,^2^ whereas a subset of ssRMSs seem to be more associated with *MYOD1* mutations; however, this particular group is evolving as we speak.^3,4^ Recent studies have made evident that *MYOD1* mutations are not restricted to FN-RMS with spindle cell morphology,^2^ supporting the need for better risk stratification beyond *MYOD1* and *RAS* mutation status. Given that the most recurrent genomic alterations in FN-RMS are copy number gains and losses,^5^ we sought to investigate the prognostic role of chromosomal aberrations in RMS.

**CONTEXT**

**Key Objective**
The genetic landscape of fusion-negative (FN) rhabdomyosarcoma (RMS) is only partially understood. In particular, the relevance of copy number aberrations in rhabdomyosarcoma genesis and their correlation with prognosis have not been fully elucidated.
**Knowledge Generated**
Chromosome (chr) 8 gain was associated with a slightly improved prognosis. By contrast, loss of chr3q was associated with worse outcome in FN-RMS compared with that in fusion-positive-RMS.
**Relevance**
Identifying copy number variations including chr3q loss or chr8 gain in FN RMS may be important for risk stratification. Future studies studying genes located on these chromosomes are needed to discover additional therapeutic targets.

## METHODS

### Study Cohort

This study was approved by the institutional review board (IRB; WUSTL 201605139). Copy number profiling data provided by the National Cancer Institute (NCI) were used for the purposes of this study. The data originated from a custom hybrid-based capture sequencing assay performed on a collection of samples from two large cohorts: UK samples were from patients treated on the malignant mesenchymal tumor and RMS2005 trials with local and national ethical approvals for analyses (CCR2015 and 06/MRE04/71, respectively). Children's Oncology Group samples were from ARST0331, ARST0431, D9602, D9803, and D9902. Publicly available data used in this work included samples collected on IRB-approved clinical trials or tissue banking studies, and all necessary patient/participant consent has been obtained. After sequencing, copy number profiling of 641 cases of FP-RMS and FN-RMS samples was performed using both on-target and off-target sequencing reads using open-source CNVKit software. The full methods are outlined in the study by Shern et al.^2^ The corresponding clinical information (sex, age, race, ethnicity, survival information, and life status), pathologic information (RMS risk group, metastasis information, anatomic group, and primary site), fusion status, and mutation status were obtained and collated. Nine cases of FN-RMS (three cases of ssRMS, six cases of ERMS) with available tissue were retrieved from the institutional archive to perform additional testing.

### Copy Number Analysis

Copy number profiling data were provided by the NCI and approved by the UK study lead for the purposes of this study. Copy number calls for each segment were calculated using the CNVKit call function.^6^ The software rounds the log_2_ ratios for each segment to the nearest absolute copy number integer given a normal diploid chromosome. Numeric log_2_ thresholds were manually set to stricter thresholds to account for lack of information regarding tumor sample purity. Each segment was mapped to the corresponding Entrez gene identification and symbol using the R libraries GenomicRanges^7^ and org.Hs.eg.db.^8^ In addition, a copy number frequency plot was generated for each case using the CNVKit scatter function. The plots were manually reviewed, and cases containing excess noise and copy artifacts were subsequently excluded. Specifically, samples were evaluated for low on-target coverage (log_2_ values near or at ±20), a wide range of log_2_ copy ratios, and high variability of log_2_ copy ratios between nearby targets. In total, 61 cases were excluded (9.52%) and 580 cases (90.4%) were kept for downstream analysis.

After review of each copy number frequency plot, a custom R^9^ script was used to bin each segment to the corresponding chromosome arm and then calculate the mean copy number integer and log_2_ ratio for each arm. The cases were then collated into a master data frame and joined to the corresponding clinical information outlined above.

### Survival Analysis

Statistical analysis was performed using R software (version 3.6, R Foundation for Statistical Computing, Vienna, Austria)^9^ and associated packages. Before analysis, copy number calls were trichotomized into copy number loss (<2 copies), copy neutral (2 copies), or gain (>2 copies) for every whole chromosome and each individual chromosome arm. Using the Survival^10,11^ and survminer libraries, univariate Cox's proportional hazard regression analysis was performed for the following predictor variables: age, RMS risk stratification, *FOXO1* fusion status (fusion-positive [FP] *v* FN), and the trichotomized copy number data. All predictor variables demonstrating a statistical significance association with survival (*P* < .05) were retained for further analysis.

To minimize overfitting and to account for likely multicollinearity between predictor variables, multivariable Cox regression was performed in combination with Least Absolute Shrinkage and Selection Operator Regression (Cox LASSO) using the Glmnet library.^12^ The Cox LASSO was bootstrapped (5,000 replicates) to account for variance in predictor variable penalization and to generate hazard ratios (HRs) with 95% bias-corrected and accelerated bootstrap confidence intervals. For each replicate/iteration, tenfold cross-validation was performed to extract the regularization parameter, lambda, which minimized coefficient of variation error plus one standard error (lambda 1se). Finally, the nonpenalized predictor variables with statically significant confidence intervals were retained and reapplied to a multivariable Cox regression (survival library) to obtain nonpenalized HRs and *P* values.

### Fluorescence In Situ Hybridization

The departmental archive at the Washington University School of Medicine was searched for cases of well- and dedifferentiated liposarcoma (WDLPS and DDLPS), leiomyosarcoma (LMS), neurofibromatosis (NF) type 1—malignant peripheral nerve sheath tumor (MPNST), non-NF1 MPNST, pleomorphic RMS (PRMS), ssRMS, and ERMS with available tumor. Centromeric enumeration (Vysis CEP8 D8Z2) was performed, and 200 cells per tumor were counted. Average of either chromosome (chr) 8 gain or chr3 loss, percentage of cells with chr8 gain or chr3 loss, and highest copy number of chr8 were assessed.

Fluorescence in situ hybridization (FISH) analysis was performed in the Clinical Cytogenetics and Molecular Pathology Laboratory. The formalin-fixed, paraffin-embedded unstained slides were baked, deparaffinized, and subject to protease pretreatment with the Paraffin Pretreatment 2 kit (Abbott Molecular Inc, Des Plaines, IL) and codenaturation. The slides were hybridized with the FISH probe set overnight, washed, and counterstained with 4,6, diamidino-2-phenylindole. Orange-deoxyuridine-triphosphatase–labeled RP11-54L9 FISH probes were used to target the 3q28 region. Green-deoxyuridine-triphosphatase–labeled chr3 centromere control probes were used to target the centromere region of chr3. A total of 200 nuclei cells were counted on each specimen.

## RESULTS

### Fluorescence In Situ Hybridization Studies Show That Chr8 Gain Is Highly Prevalent in FN-RMS

**FIG 1.**
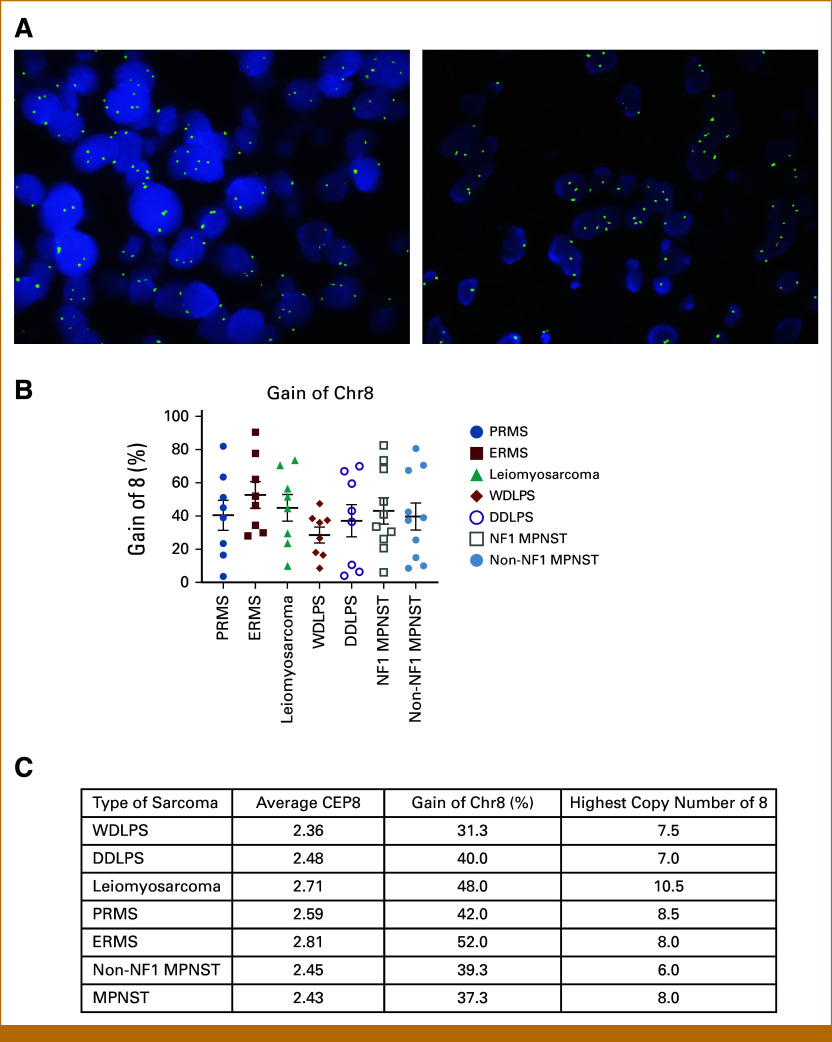
Chr8 gain is a common event in soft tissue sarcoma. (A) Left and right images demonstrate two cases of ERMS showing more than two signals per nucleus. (B) The graph demonstrates the gain of chr8 in percent per soft tissue sarcoma. (C) The table outlines the average chr8, the gain of chr8 in percent, and the highest copy number of chr8 per cell in the tested group of soft tissue sarcomas. Chr, chromosome; DDLPS, dedifferentiated liposarcoma; ERMS, embryonal RMS; MPNST, malignant peripheral nerve sheath tumor; NF, neurofibromatosis type; PRMS, pleomorphic RMS; RMS, rhabdomyosarcoma; WDLPS, well-differentiated liposarcoma.

We previously showed that chr8 gain is associated with overall worse outcomes in patients with soft tissue sarcomas (STS)^13^ and therefore sought to study the importance of chr8 gain in a cohort of the most common STS. Sixty-five samples were subjected to chr8 FISH: 13 cases of ERMS, eight cases of PRMS, eight cases of LMS, eight cases of WDLPS and their eight corresponding DDLPS, 10 cases of NF1-MPNST, and 10 cases of non-NF1 MPNST. The highest chr8 gain was noted in ERMS with an average copy number of 2.8 copies, followed by LMS with 2.7. ERMS showed chr8 gain in more than half of the counted cells per sample (average, 52%; range, 28%-59; Fig 1A). The highest individual copy number per cell was seen in LMS with an average of 10.5 copies, followed by 8.5 copies in PRMS (range, 6.5-15.1) and 8.0 copies in ERMS. There was no difference in copy number gain between WDLPS and its corresponding DDLPS (*P* = .7, .47 and .40, respectively; Fig 1B). These data support that chr8 gain is a highly prevalent copy number variation (CNV) in STS in general, with ERMS being the most common in our tested cohort.

### CNV Profiling Data

Next, we analyzed the CNV profiling data of the 641 cases of FP-RMS and FN-RMS provided by the NCI. After review of the frequency plots, 580 cases (90.5%) were kept for additional analysis. Overall clinical details of these cases are characterized in the study by Shern et al.^2^ In brief, patients presented with tumors in the parameningeal site (20%), paratesticular region (20%), retroperitoneum or trunk (16%), and extremities (14%). The male to female ratio was 1.9:1, and the median age was 5.9 years (range, 0.02-37.8 years). Similar to previous reports, we confirmed the importance of fusion status on overall survival (OS) with FP cases having worse OS (HR of 3.711; *P* < .001) compared with FN cases (Data Supplement, Fig S1 and Table S2). In addition, we confirmed that the intermediate and low risk groups had better overall survival compared to the high risk group (0.375; *P* ≤ .001; 0.127, *P* < .001).

### Chr8 Gain Is Associated With Improved Prognosis in FN-RMS

**FIG 2.**
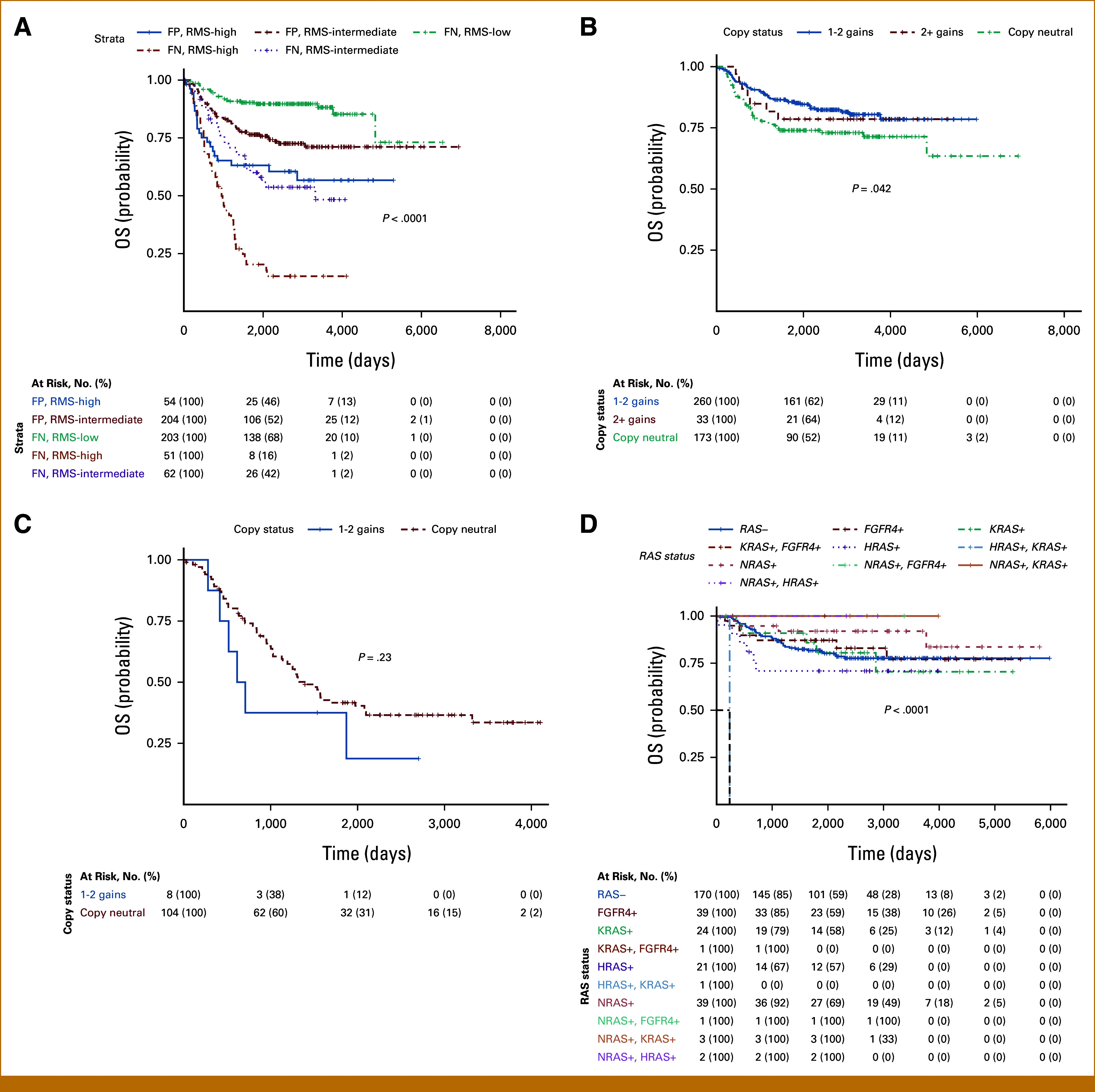
Effects of chr8 gain on OS. (A) Fusion status and risk stratification are significant for OS. (B) Gains in chr8 are associated with improved OS in fusion-negative cases. (C) No statistically significant association with gains in chr8 in fusion-positive cases. (D) OS similar regardless of the presence or absence of *RAS* pathway gene mutation. Chr, chromosome; FN, fusion-negative; FP, fusion-positive; OS, overall survival; RMS, rhabdomyosarcoma.

Similar to that identified by FISH on our internal cohort, a gain in chr8 was identified in 301 (51.9%) tumors with eight (2.7%) and 293 (97.3%) of these being FP or FN, respectively. Next, we analyzed the correlation between chr8 gain and OS. We found that gains in chr8 correlated with a small improvement in OS in FN-RMS cases (*P* = .042; Fig 2B) but not FP-RMS cases (*P* = .23; Fig 2C). We additionally examined whether the presence of a mutation in a RAS pathway gene (*KRAS*, *HRAS*, *NRAS*, and *FGFR4*) had an association with OS in cases with chr8 gains (Fig 2D).

Overall cases with RAS pathway gene mutations had similar OS with the exception of one case with a mutation in both *HRAS* and *KRAS* (OS, 235 days).

Finally, we examined whether other copy number alterations detailed in the evolutionary model proposed by Chen et al^14^ influenced OS. We were not able to demonstrate any statistically significant prognostic/survival changes for cases that demonstrated gains in chr9, chr12p, or chr13q. In addition, we did not identify significant survival differences in cases carrying any combination of the described copy number changes, including cases harboring gains in chr8 (Data Supplement, Fig S1).

### Loss of Chr3q Associated With Decreased OS

Using the 580 cases from NCI, we performed univariate cox proportional hazard analysis for every whole chromosome and each chromosome arm (p and q-arms; Data Supplement, Table S1). Of the 68 possible variables, 31 demonstrated statistically significant associations with OS (Data Supplement, Table S2). When restricted to only FN cases, 22 variables remained statistically significant, with all variables demonstrating worse OS (HR >1; Data Supplement, Table S3; Table 1). After feature selection (Data Supplement, Table S4) and multivariable analysis, gain of chr8q and loss of chr3q remained as the only statistically significant variables associated with OS. Gain in chr8q demonstrated better OS (HR, 0.63; *P* = .028), and loss of chr3q demonstrated worse OS (HR, 2.99, *P* = .048; Data Supplement, Table S3; Table 2).

**TABLE 1.**
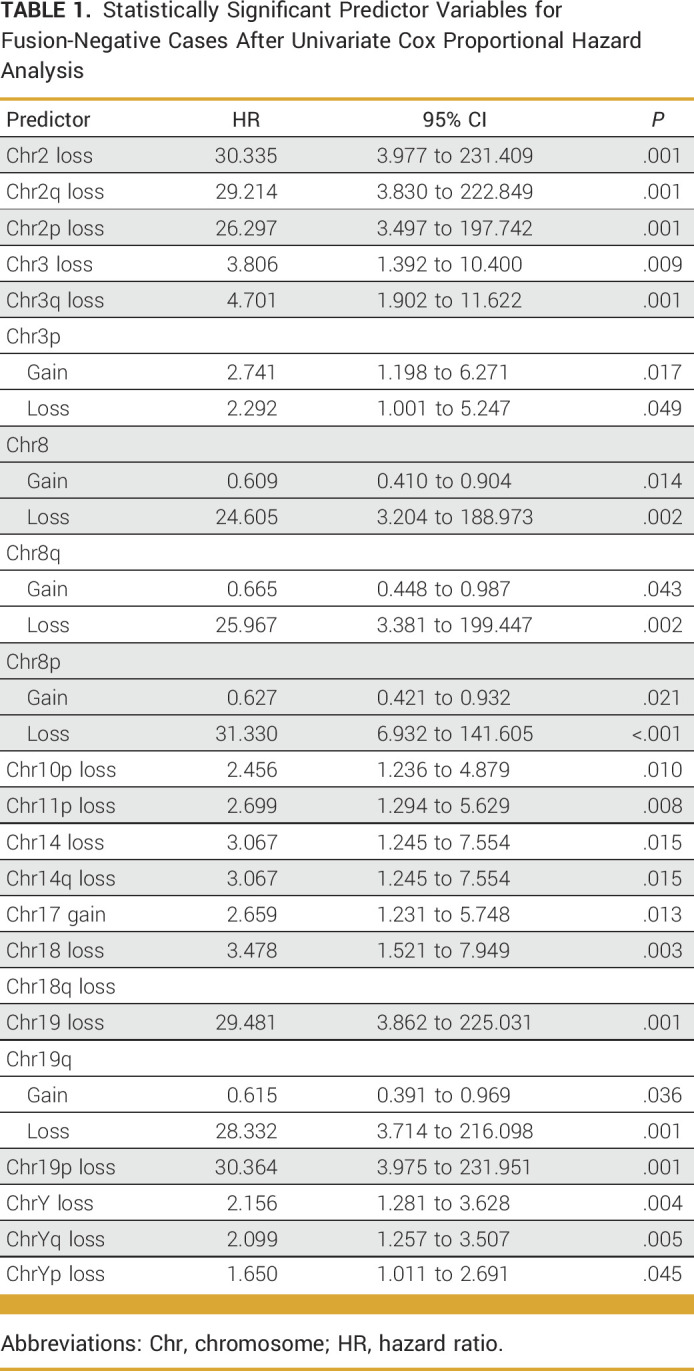
Statistically Significant Predictor Variables for Fusion-Negative Cases After Univariate Cox Proportional Hazard Analysis

**TABLE 2.**
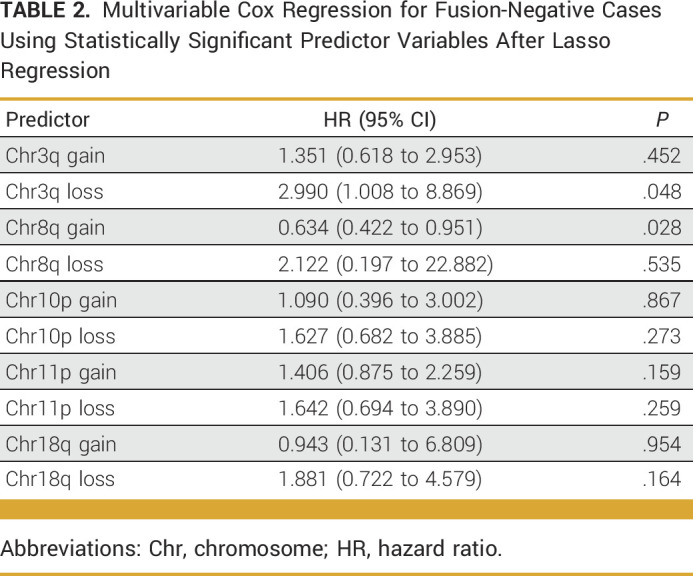
Multivariable Cox Regression for Fusion-Negative Cases Using Statistically Significant Predictor Variables After Lasso Regression

**FIG 3.**
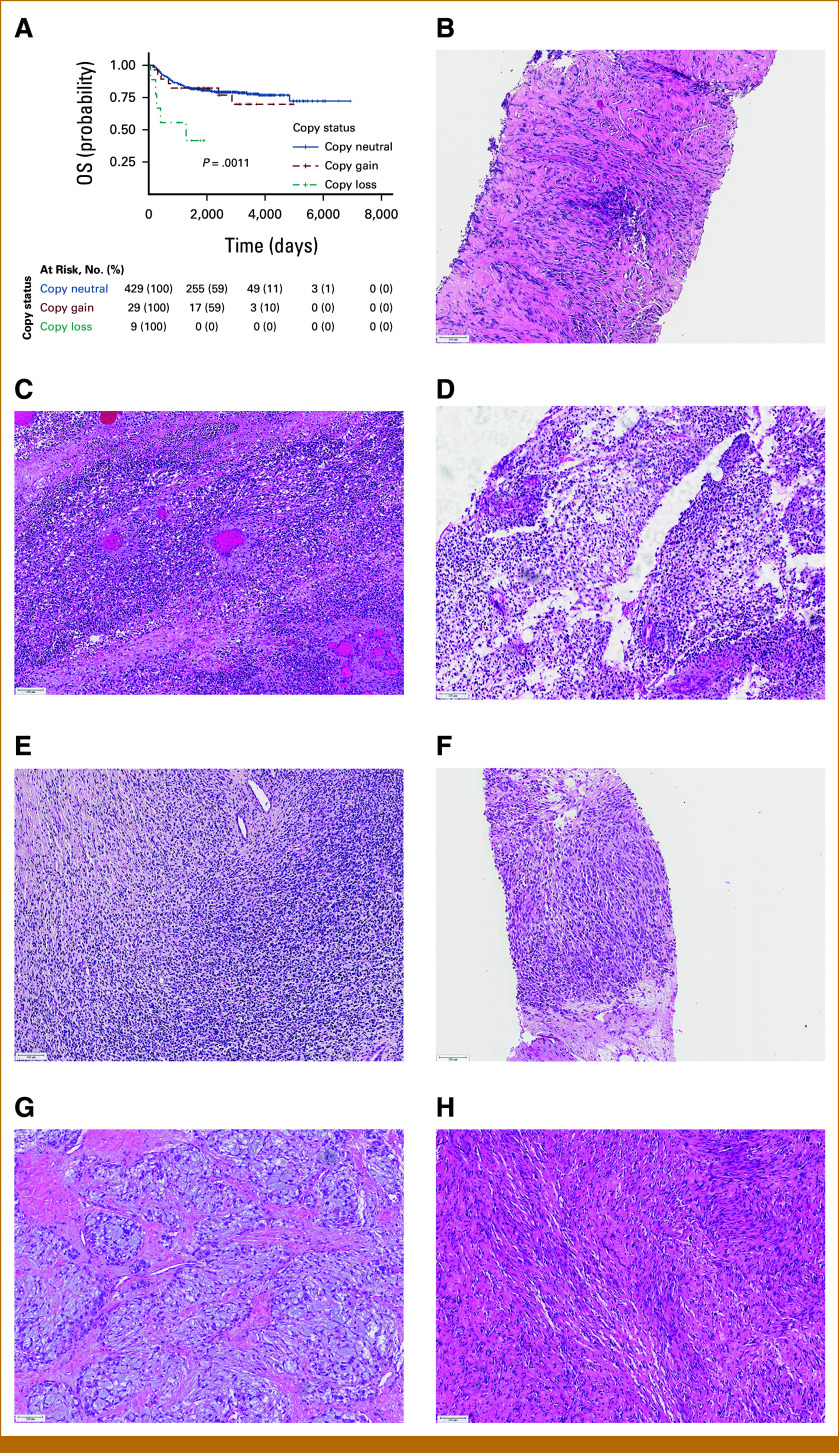
Loss of chr3q is associated with worse overall survival in FN-RMS. (A) Graph outlining the finding that chr3q loss leads to worse outcome. (B-H) Histology of cases showing 3q loss. Chr, chromosome; FN, fusion-negative; RMS, rhabdomyosarcoma.

Overall, these findings suggest that a loss of chr3q is associated with worse OS in FN cases of RMS. Similar analysis was performed on FP cases but is out of scope for the purpose of this article (Data Supplement, Tables S5 and S6). More specifically, of the 467 FN cases (80.5%), nine cases demonstrated loss of the q-arm of chr3 (1.9%). Available histologic sections of seven of these nine tumors were subsequently evaluated. These cases demonstrated a heterogenous morphology with four demonstrating spindle cell morphology, suggestive of ssRMS, whereas three showed a predominately primitive round cell morphology suggestive of ERMS (Fig 3). As *MYOD1* mutation is known as an independent poor prognostic factor, we assessed all nine cases for *MYOD1* mutations. Cases RMS2248 and RMS2505 showed a *MYOD1* mutation, both of which had spindle cell morphology, whereas the remaining seven tumors did not.

### Chr3q Validation

**FIG 4.**
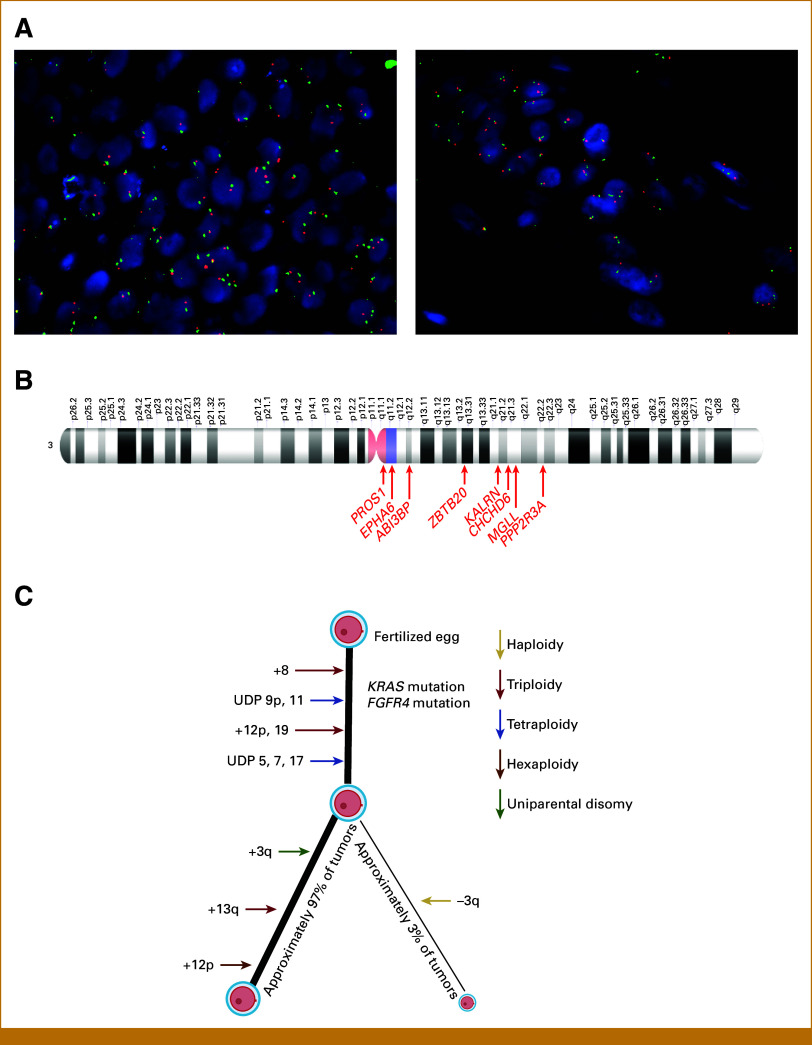
Chr3 in FN-RMS. (A) Representative images of a cohort of ssRMS and ERMS show monosomy of chr3. (B) Top hit cancer related to chr3q after correlation with RMS cell lines. Chr, chromosome; ERMS, embryonal RMS; FN, fusion-negative; RMS, rhabdomyosarcoma; ssRMS, spindle cell/sclerosing RMS.

We attempted to examine whether loss of chr3q was present in a small cohort of internal departmental cases, which consisted of six ERMSs (previously assessed for chr8 gain by FISH, see above) and added three cases of ssRMS. While none of the cases demonstrated complete or partial loss of just the q-arm, a subset of cases (one case of ssRMS, four cases of ERMS) did demonstrate complete loss of one copy of chr3 (Fig 4A; Data Supplement, Table S9). Overall, this finding is consistent with that of the NCI data set analysis given that a loss of one copy of chr3 (defined as log_2_ <–0.4) was found in eight of nine FN cases with loss of chr3q.

Next, using the mapped Entrez gene identifications, we investigated all possible cancer-related genes present on the q-arm of chr3. We then correlated these with the genomic data of the three FN-RMS cell lines (BIRCH, CTR, and RMS-YM). Overall, there were 33 cancer-related genes (Data Supplement, Table S7), including *ABI3BP*, *CHCHD6*, *EPHA6*, *KALRN*, *KBTBD12*, *MCM2*, *MGLL*, *PPP2R3A*, *PROS1*, *FGF12*, *MECOM*, *SERPINI1*, *MED12L*, and *ZBTB20* (Fig 4B; Data Supplement, Table S8), lost in all nine cases of RMS and lost in at least two of the three tested cell lines. All nine RMS cases showed loss of these genes in at least two of three cell lines tested.

On the basis of these data, we suggest that a small percentage of tumors (3% in our cohort; Fig 4C) show loss of chr3q, which may lead to loss of important tumor suppressor genes or other antitumoral genes and could promote tumor progression explaining the worse outcome in these cases.

## DISCUSSION

Over the recent years, genetic profiling of STS using next-generation sequencing (NGS) has become a crucial tool not only for diagnosis but also for risk stratification of these tumors. Multiple tumor subtypes are now identified by histologic appearance in combination with a characteristic fusion, driver mutation, or the presence of a certain copy number profile. The first molecular tools for substratification of RMS came with the definition of the fusion of *FOXO1* with *PAX3* or *PAX7*, which splits tumors into two major subtypes and roughly correlates with the histologic features: ERMS and alveolar RMS.^15,16^ More recently, data suggest that some of the previously categorized ERMS tumors have a distinctive morphology composed of elongated spindle cells and often sclerotic stroma. The WHO classification since 2013^16,17^ has classified these tumors as ssRMS with a subset of them carrying mutations in *MYOD1*.

Nonetheless, given the known imprecision of the current risk stratification particularly for those with intermediate-risk FN-RMS, additional methods such as CNV analysis are necessary to risk stratify this large and heterogenous group of tumors.^14^

We assessed the role of common CNVs such as chr8 gain in FN-RMS and other copy number changes described in the evolution of FN-RMS aiming to assess their prognostic relevance. Our data showed that chr8 gain, as previously shown,^18^ is a common CNV in ERMS. This is not surprising as trisomy of chr8 was commonly an early event in rhabdomyosarcomagenesis^14^ and may be important for tumor initiation. Interestingly, however, our analysis demonstrated that a gain of chr8 in FN-RMS was associated with a slight improvement in OS compared with copy neutral cases. A similar finding was also observed after proportional hazard modeling, which demonstrated that a gain in chr8q was associated with better OS. This finding is in contrast to the majority of STS where chr8 gain is more often associated with worse OS.^13^ There are a few studies on nonmesenchymal tumors including prostate and breast cancers in which loss of chr8 confers a worse prognosis,^19,20^ so one may argue that subsequent gain of chr8 may help to rescue this effect. Additional studies will be needed to further substantiate those findings.

Meanwhile, evaluation of other chromosomes that have been reported as aneuploidy events was not found to be associated with OS. The one exception was loss of the q-arm of chr3, which was associated with significantly worse outcomes in a small subset of FN-RMS. This finding is different from that reported in the study by Chen et al,^14^ which reported up to 50% of events containing trisomies of chr12, chr13, chr19, or chr20; hexasomy of chr12p; uniparental disomy of chr5, chr7, or chr17; and, interestingly, tetrasomy of chr3 over the course of a molecular lifetime. No case in that work demonstrated the presence of a loss of chr3q, highlighting the relative rarity of this genomic alteration in the population. Given the generally favorable outcome for the majority of patients with FN-RMS, it is possible that this is the molecular path the majority of FN-RMS follow.

We also attempted to determine the presence of chr3q loss in an internal cohort of nine cases using FISH. Although we did not detect loss of only the q-arm of chr3 in any of the cases, we were able to detect a monosomy of chr3 in four of the nine cases. Importantly, although limited available follow-up did not allow for significant findings on outcome analysis, one of the chr3 monosomy patients died within 1 year of diagnosis and another patient suffered from a local recurrence just 13 months after diagnosis. This supports the possibility that loss of chr3q might have a negative effect on OS. Additional follow-up of these patients is needed, however, for a more definitive determination. Interestingly, two of the nine cases showing chr3q loss had a *MYOD1* mutation, a variant associated with poor outcomes in itself^3,4^; it remains unclear how these two findings relate to each other if at all.

These results raise the obvious question of which gene(s) or specifically the loss of which gene(s) contributes to tumor progression. Comparing the genomic data of three FN-RMS cell lines with our cohort of tumors, we found several candidate genes (Fig 3) in which loss has been associated with tumor progression in other neoplasms.^21,22^ For instance, loss of *PROS1* was found to be associated with metastatic disease in uveal melanoma by broad copy number analysis, together with loss of *CCDC50*.^23^ In addition, osteosarcoma has been shown to have loss of chr3q among many chromosomal aberrations,^24^ specifically loss of the tumor suppressor gene limbic system–associated membrane protein, which usually reduces the proliferation of normal osteoblasts by regulation of apoptotic and cell cycle transcripts.^25^

Mechanistic studies will be needed to elucidate the functional and prognostic relevance of these genes. In addition, we may hope that the Molecular Characterization Initiative as part of NCI's Childhood Cancer Data Initiative, which is currently working on performing large-scale NGS on any childhood STS, provides stimulating data to propel exciting times in this rapidly evolving field.

In conclusion, the pathogenesis of STS is poorly understood and many are associated with poor outcome, supporting the need for new treatment approaches with better risk stratification. Our data suggest that loss of chr3q is associated with poor OS in FN-RMS, and future work aims to understand the functional relevance of this chromosomal alteration in RMS.
